# New Method to Estimate Central Systolic Blood Pressure From Peripheral Pressure: A Proof of Concept and Validation Study

**DOI:** 10.3389/fcvm.2021.772613

**Published:** 2021-12-15

**Authors:** Denis Chemla, Sandrine Millasseau, Olfa Hamzaoui, Jean-Louis Teboul, Xavier Monnet, Frédéric Michard, Mathieu Jozwiak

**Affiliations:** ^1^Service d'explorations fonctionnelles multidisciplinaires bi-site Antoine Béclère - Kremlin Bicêtre, GHU Paris Sud, AP-HP, Le Kremlin-Bicêtre, France; ^2^Université Paris-Sud, Faculté de Médecine, Université Paris-Saclay, Le Kremlin-Bicêtre, France; ^3^INSERM UMR_S 999, Hôpital Marie Lannelongue, Le Plessis Robinson, France; ^4^Pulse Wave Consulting, Saint Leu La Foret, France; ^5^Service de Réanimation Polyvalente, Hôpital Antoine Béclère, Hôpitaux Universitaires Paris-Sud, Assistance Publique-Hôpitaux de Paris, Clamart, France; ^6^Service de Médecine Intensive-Réanimation, Hôpital Bicêtre, Hôpitaux Universitaires Paris-Sud, Assistance Publique-Hôpitaux de Paris, Le Kremlin-Bicêtre, France; ^7^MiCo, Denens, Switzerland; ^8^Equipe 2 CARRES, UR2CA - Unité de Recherche Clinique Côte d'Azur, Université Côte d'Azur UCA, Nice and Service de Médecine Intensive Réanimation, Centre Hospitalier Universitaire l'Archet, Nice, France

**Keywords:** aortic pressure, central systolic blood pressure, mean blood pressure, diastolic blood pressure, cardiovascular risk

## Abstract

**Objective:** The non-invasive estimation of central systolic blood pressure (cSBP) is increasingly performed using new devices based on various pulse acquisition techniques and mathematical analyses. These devices are most often calibrated assuming that mean (MBP) and diastolic (DBP) BP are essentially unchanged when pressure wave travels from aorta to peripheral artery, an assumption which is evidence-based. We tested a new empirical formula for the direct central blood pressure estimation of cSBP using MBP and DBP only (DCBP = MBP^2^/DBP).

**Methods and Results:** First, we performed a *post-hoc* analysis of our prospective invasive high-fidelity aortic pressure database (*n* = 139, age 49 ± 12 years, 78% men). The cSBP was 146.0 ± 31.1 mmHg. The error between aortic DCBP and cSBP was −0.9 ± 7.4 mmHg, and there was no bias across the cSBP range (82.5–204.0 mmHg). Second, we analyzed 64 patients from two studies of the literature in whom invasive high-fidelity pressures were simultaneously obtained in the aorta and brachial artery. The weighed mean error between brachial DCBP and cSBP was 1.1 mmHg. Finally, 30 intensive care unit patients equipped with fluid-filled catheter in the radial artery were prospectively studied. The cSBP (115.7 ± 18.2 mmHg) was estimated by carotid tonometry. The error between radial DCBP and cSBP was −0.4 ± 5.8 mmHg, and there was no bias across the range.

**Conclusion:** Our study shows that cSBP could be reliably estimated from MBP and DBP only, provided BP measurement errors are minimized. DCBP may have implications for assessing cardiovascular risk associated with cSBP on large BP databases, a point that deserves further studies.

## Introduction

In the recent years, there has been a growing interest toward improving cardiovascular risk estimation using central (aortic root) blood pressure (BP) (1, 2). It is the central BP which loads the heart, and there is an anatomic proximity of the aorta to the brain and kidneys. Thus, end-organ damages due to pressure overload and cardiovascular complications may be more closely related to central than peripheral BP (3–6), although this point remains debated (7–9). The peripheral systolic BP (SBP) is most often higher than central systolic BP (cSBP,) and this pressure amplification is mainly explained by the narrowing of arterial caliber and by arterial properties, especially arterial stiffness which affects the speed of the pressure pulse wave traveling down from the heart to periphery and back to the heart, thus impacting the amount of both pressure wave amplification and reflection (1, 2, 10–19). As a result, peripheral SBP is considered an inaccurate substitute of cSBP. On the other hand, the mean BP (MBP) recorded in peripheral large arteries only slightly differ from central aortic value in a supine subject, and the same applies to diastolic BP (DBP) (1, 2, 10–20).

Invasive recordings at the central level provide the gold-standard measure of true aortic root cSBP, but they are limited to patients requiring catheterization and thus not ethically nor technically feasible in the general population. There has been an ongoing development of new devices to non-invasively estimate cSBP using various waveform acquisition techniques (tonometry, oscillometry, and echo-tracking) (20–27). Some devices allow a calibration method based on brachial cuff SBP and DBP. However, studies carried out nowadays most often rely on the widely accepted assumption that peripheral MBP and DBP may be used as input values for central MBP and DBP (21, 22, 25). A logical implication is that any empirical equation allowing the accurate and precise estimation of cSBP from central MBP and DBP would theoretically also allow the estimation of cSBP from peripheral MBP and DBP without the need for any supplemental device to record waveforms. In the remaining part of our manuscript, MBP and DBP will interchangeably refer to central or peripheral BP values, except where indicated.

**Figure 1:**
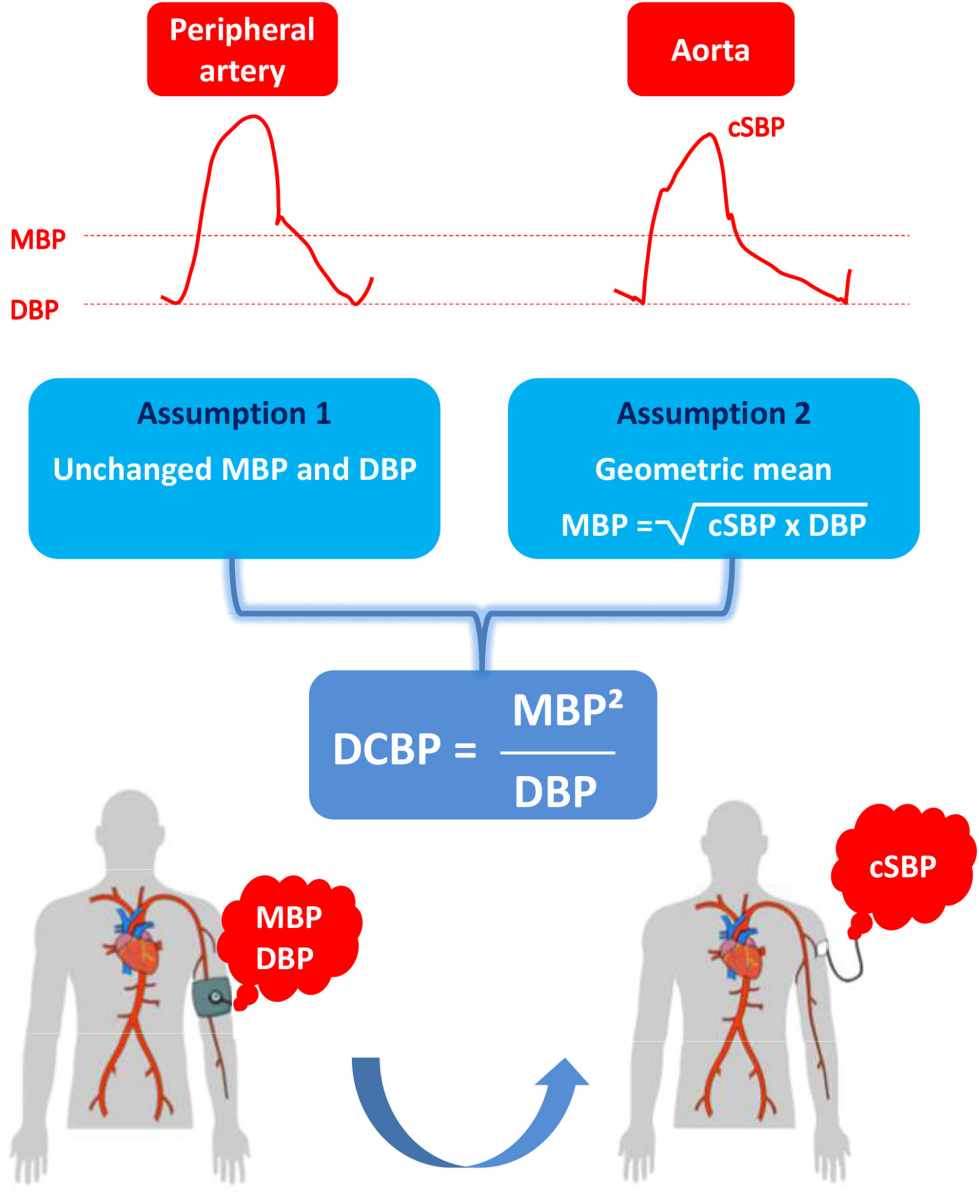
Rationale of the DCBP formula. cSBP, central systolic blood pressure; DBP, diastolic blood pressure (either central or peripheral); MBP, mean blood pressure (either central or peripheral); DCBP, direct central blood pressure estimation of csBP.

Based on the basic principles of hemodynamics, here we propose a new formula for the direct central blood pressure estimation of cSBP, which is DCBP = MBP^2^/DBP (see Methods and Figure 1). Our proof-of-concept and validation study tested the accuracy and precision of DCBP using invasive MBP and DBP values in an attempt to minimize BP measurement errors, as previously recommended (21–25).

## Methods

### Derivation of the DCBP Formula

Our group (28–30) and others (31) have proposed various empirical formulas relying on aortic SBP and DBP to estimate MBP at the aortic root level. In a previous high-fidelity aortic pressure study (28), we showed that the geometric mean of aortic SBP and DBP provides an accurate and precise estimate of the time-averaged aortic MBP:

Aortic MBP = $\sqrt{\text{aorticSBP}\times aorticDBP}$. This equation may be rewritten as follows:

Aortic MBP^2^ = aortic SBP × aortic DBP

A new empirical formula may thus be proposed to estimate central (aortic) SBP:

DCBP = aortic MBP^2^/aortic DBP

The first aim of our study was to establish the proof-of-concept by studying the accuracy and precision of this DCBP formula at the aortic root level.

The same DCBP formula may well also apply at the peripheral (brachial and radial) level, given that MBP and DBP values only slightly differ as pressure travels from aorta to brachial and radial artery**:**

DCBP = brachial MBP^2^/brachial DBP

DCBP = radial MBP^2^/radial DBP

The second aim of our study was to validate these DCBP formulas using MBP and DBP values that were invasively obtained at the brachial and radial artery level.

### DCBP at the Aortic Root Level Using High-Fidelity Pressure Recordings (Proof of Concept, *n* = 139)

The DCBP formula was tested using a *post-hoc* analysis of our prospective high-fidelity aortic pressure database (28). All investigations had been approved by our institution, and informed consent was obtained for all patients. The adult patients prospectively enrolled were free of aortic stenosis or left ventricular outflow tract obstruction. The underlying diagnosis was as follows: subjects with normal cardiac function and coronary angiograms (*n* = 31), subjects with known hypertension (*n* = 46), grafted hearts (*n* = 18), idiopathic dilated cardiomyopathy (*n* = 14), and miscellaneous cardiac diseases, mainly coronary artery disease (*n* = 30). The invasive BP, namely the time-averaged MBP, cSBP, and DBP were automatically measured.

### Validation of DCBP Using Invasive Peripheral BP

#### Invasive High-Fidelity Aortic and Brachial Pressures (*n* = 64)

A recent systematic review by our group (19) has documented that only two independent high-fidelity pressure studies have reported the full invasive BP data set (SBP, DBP, and MBP), either simultaneously or during the same overall procedure at both the aortic root and brachial artery level (10, 11). These studies were used to test the accuracy and precision of the DCBP formula (*n* = 64). As also previously discussed (19), two studies were excluded from the analysis because patients were given nitroglycerin (12) or because the data presented (13) have been subsequently upgraded (11), and four studies were excluded because they documented aortic/brachial SBP and DBP only, not MBP (14–17).

#### Carotid Tonometry Coupled With Invasive Fluid-Filled Radial BP (*n* = 30)

To prospectively validate the DCBP formula, we conducted an observational study in the 25-bed medical ICU of a University hospital. The study was approved by the Ethics Committee of the French Intensive Care Society (agreement 12-376). All patients or their next of kin were informed and consented to participate. We included 30 consecutive spontaneously breathing patients who were hemodynamically stable and already equipped with a fluid-filled catheter in the radial artery. Only spontaneously breathing patients were included to ensure high-quality pressure signal of carotid tonometry (32). Exclusion criteria were heart rate >120 bpm, aortic stenosis or obstructive cardiomyopathy, medical history of carotid occlusive diseases, or carotid murmur at auscultation. Carotid tonometry (Complior Analyse® ALAM Medical, Saint-Quentin-Fallavier, France) was performed, as previously described (23, 32, 33). The advantage of using carotid tonometry relies on the fact that no transfer function or peripheral waveforms analysis is used to estimate cSBP (33). Briefly, we obtained non-invasive carotid pressure on the same side as the arterial catheter, except in patients with a central venous catheter in the superior vena cava territory, in whom we studied the contralateral carotid to avoid any potential artifact. The carotid tonometer pressure signals were calibrated from the invasive radial DBP and the time-averaged radial MBP (input factors), assuming unchanged DBP and MBP from aorta to the peripheral arteries. Carotid SBP was used as a non-invasive estimate of cSBP, an assumption which is evidence-based (20, 34). In all patients, non-invasive central BP (carotid tonometry) and invasive peripheral BP (radial artery catheter) were recorded simultaneously.

### Sensitivity Analysis

The theoretical influences of isolated or combined (parallel or opposite) calibration errors in DBP or MBP on the DCBP-derived estimation of cSBP were studied. The following example illustrates the way calculations were performed: as compared to intra-arterial BP values, a +5% overestimation of MBP together with a +10% overestimation of DBP resulted in a +0.2% overestimation of cSBP by the formula DCBP [(1.05 × 1.05)/1.10 =1.0025]. The DCBP-derived estimates of cSBP were deemed reasonably acceptable from a clinical standpoint if the associated percentage error ranged from −10 to +10%.

### Statistical Analysis

The normal distribution of data was checked by the Kolmogorov–Smirnov test. Continuous variables were summarized as mean ± standard deviation (SD) or median [interquartile range], and categorical variables as counts (percentages). Correlations between variables were assessed by Pearson's correlation coefficient. Comparison between men and women were performed using Student's unpaired t-test. The error was calculated as the difference between DCBP and cSBP and was expressed in mmHg. The accuracy (mean error) and precision (SD) of DCBP estimate were calculated. As previously recommended (35), the difference was categorized into four bands according to its rounded absolute value: 0–5 mmHg which represent measurements considered to be very accurate (no error of clinical relevance); 6–10 mmHg, which represent measurements considered to be slightly inaccurate; 11–15 mmHg, which represent measurements considered to be moderately inaccurate; and >15 mmHg which represent measurements considered to be very inaccurate. The final analysis was based on how values in these bands fall cumulatively into three zones: within 5 mmHg (this zone represents all values falling in the 0–5 mmHg band), within 10 mmHg (this zone represents all values falling in the 0–5 and 6–10 mmHg bands), and within 15 mmHg (this zone represents all values falling in the 0–5, 6–10, and 11–15 mmHg bands). The error was also expressed as a percentage of cSBP given the high number of hypertensive patients in the study population. Statistical analysis was performed with MedCalc11.6.0 software (MedCalc, Mariakerke, Belgium), and a p-value < 0.05 was considered statistically significant.

## Results

### High-Fidelity Aortic Pressure Analysis (Proof of Concept, *n* = 139)

Patients were mostly men (78%) and were middle-aged (49 ± 12 years) (Table 1). The cSBP varied across a wide range, from 82.5 to 204.0 mmHg. Six patients (4%) had their cSBP ≤ 100 mmHg, 13 patients (9%) had cSBP between 100 and 140 mmHg, 66 patients (47%) had cSBP between 140 and 160 mmHg, and 54 patients (39%) had cSBP ≥ 160 mmHg. Thus, 120/139 (86%) patients had their cSBP ≥ 140 mmHg at the time of the catheterization. Aortic DBP ranged from 49.8 to 133.0 mmHg. Six patients (4%) had their DBP ≤ 60 mmHg, 39 patients (28%) had their DBP ≥ 85 mmHg, and 14 patients (10%) had their DBP ≥ 100 mmHg.

**Table 1:** Demographic and hemodynamic characteristics of the patients from the invasive high-fidelity aortic pressure study.

***N***	**139**
Males, *n* (%)	109 (78)
Age, years	49 (12)
Heart rate, bpm	74 (12)
DBP, mmHg	80.8 (13.8)
MBP, mmHg	107.9 (18.2)
cSBP, mmHg	146.0 (31.1)
DCBP, mmHg	145.1 (30.8)
Error, mmHg	−0.9 (7.4)
Error, % cSBP	−0.4 (5.0)

*Mean (SD) values are presented except for gender, n (%)*.

*MBP, mean aortic blood pressure; DBP, diastolic aortic blood pressure; cSBP, central systolic aortic blood pressure (systolic aortic blood pressure)*.

*DCBP (Direct Central Blood Pressure), aortic MBP^2^/ aortic DBP*.

*The error (DCBP - cSBP) and % error [100 × (DCBP - cSBP) / cSBP] were calculated from the original raw data*.

**Figure 2:**
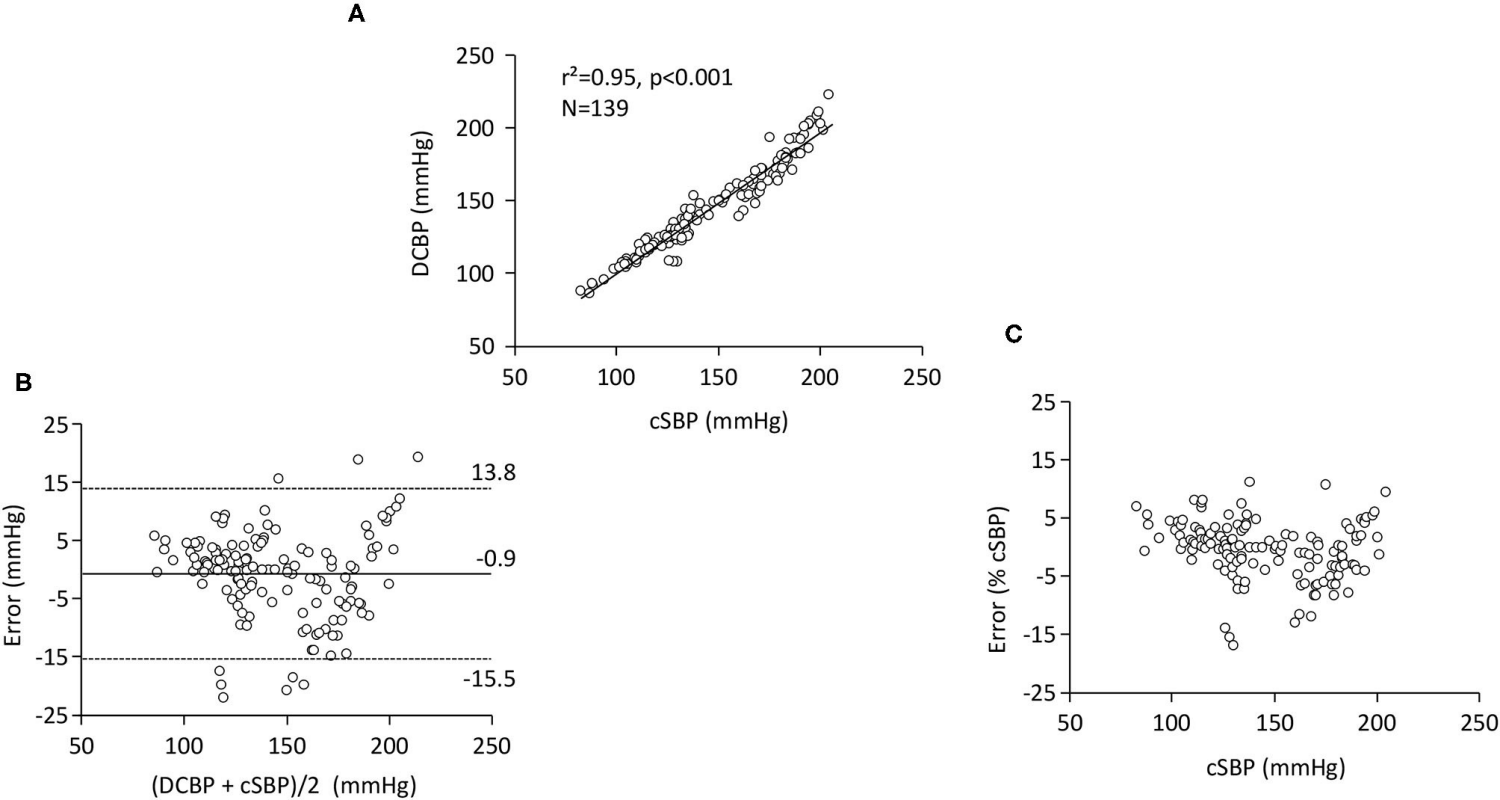
Aortic high-fidelity pressures (*n* = 139). **(A)** Linear relationship between central systolic blood pressure (cSBP) and the DCBP formula calculated from MBP and DBP. **(B)** Bland and Altman plots of the error (DCBP-cSBP) as a function of average (DCBP+cSBP)/2). Dotted lines indicate 95% limits of agreement. **(C)** Influence of cSBP on the error (expressed as a percentage of cSBP).

There was a strong linear relationship between DCBP and cSBP [r^2^ = 0.95, p < 0.001 (Figure 2A)]. As compared to cSBP (146.0 ± 31.1 mmHg), the DCBP formula gave an accurate estimate of cSBP (mean error = −0.9 mmHg), the precision was 7.4 mmHg, and there was no bias across the range (r^2^= 0.01, p = 0.69) (Figure 2B). The % error was −0.4 ± 5.0% of cSBP (range: from −16.9 to 11.3%) (Figure 2C). The number of comparisons falling within the 5, 10, and 15 mmHg error bands was 86 (61.9%), 119 (85.6%), and 130 (93.5%), respectively. The error was similar in men and women (p = 0.67), it slightly increased with heart rate (r^2^ = 0.06, p < 0.05), and it slightly decreased with age (r^2^ = 0.10, p < 0.05).

### Validation of DCBP Using Invasive Peripheral BP

#### Invasive High-Fidelity Brachial and Aortic Pressures (*n* = 64)

The invasive brachial pressures, the cSBP, and the DCBP calculated from brachial pressures are indicated in Table 2. In the two selected studies (10, 11), the weighed mean error (DCBP – cSBP) was 1.1 mmHg.

**Table 2:** Data from high-fidelity pressure studies reporting full data set of SBP, MBP, and DBP at both aortic and brachial level.

	**Kelly et al. (10)**	**Sung et al. (11)**	**Overall population**
*N*	14	50	64
Males, *n* (%)	93	74	78
Age, years	54	64	62
Brachial DBP, mmHg	71.8	70	70.4
Brachial MBP, mmHg	98.2	97	97.3
Brachial SBP, mmHg	136.3	138	137.6
cSBP, mmHg	131.3	134	133.4
DCBP, mmHg	134.3	134.4	134.5
Mean error, mmHg	3.0	0.4	1.1
Mean error, %	2.3	0.3	0.8

*Data of Kelly et al. and Sung et al. are mean values, as presented by the authors*.

*Data in the overall population are weighed means*.

*SBP, systolic blood pressure; MBP, mean blood pressure; DBP, diastolic blood pressure; cSBP, central systolic blood pressure (aortic systolic blood pressure)*.

*DCBP (Direct Central Blood Pressure), brachial MBP^2^/brachial DBP*.

*Error, DCBP — cSBP*.

*% error = 100 × (brachial DCBP - cSBP) / cSBP*.

#### Carotid Tonometry Coupled With Invasive Fluid-Filled Radial BP (*n* = 30)

Patients were mostly men (77%), with a mean age of 62 ± 14 years and a median body mass index of 22 (19–26) kg/m^2^ (Table 3). Two patients (7%) had atrial fibrillation and 10 patients (33%) received norepinephrine with a median dosage of 0.13 [0.11–0.30] μg/kg/min. The carotid cSBP ranged from 67 to 154 mmHg. Seven patients (23%) had their cSBP ≤ 100 mmHg, 21 patients (70%) had their cSBP between 100 and 140 mmHg, and two patients (7%) had their cSBP ≥ 140 mmHg. The carotid DBP ranged from 41 to 90 mmHg. Fifteen patients (50%) had their carotid DBP ≤ 60 mmHg, only one patient (3%) had carotid DBP ≥ 85 mmHg.

**Table 3:** Demographic and hemodynamic characteristics of the intensive care unit patients equipped with a fluid-filled catheter in the radial artery.

***N***	**30**
Males, *n* (%)	23 (77)
Age, years	62 (14)
Heart rate, bpm	83 (13)
Radial DBP, mmHg	60.9 (9.9)
Radial MBP, mmHg	83.5 (11.6)
Radial SBP, mmHg	128.6 (19.6)
cSBP, mmHg	115.7 (18.2)
DCBP, mmHg	115.3 (18.3)
Error, mmHg	−0.4 (5.8)
Error, % cSBP	−0.2 (4.8)

*Mean (SD) values are presented except for gender, n (%)*.

*SBP, systolic blood pressure; MBP, mean blood pressure; DBP, diastolic blood pressure; cSBP, central systolic blood pressure non-invasively estimated by carotid tonometry*.

*DCBP (Direct Central Blood Pressure), radial MBP^2^/radial DBP*.

*Error, DCBP — cSBP*.

*% error = 100 × (DCBP - cSBP) / cSBP*.

**Figure 3:**
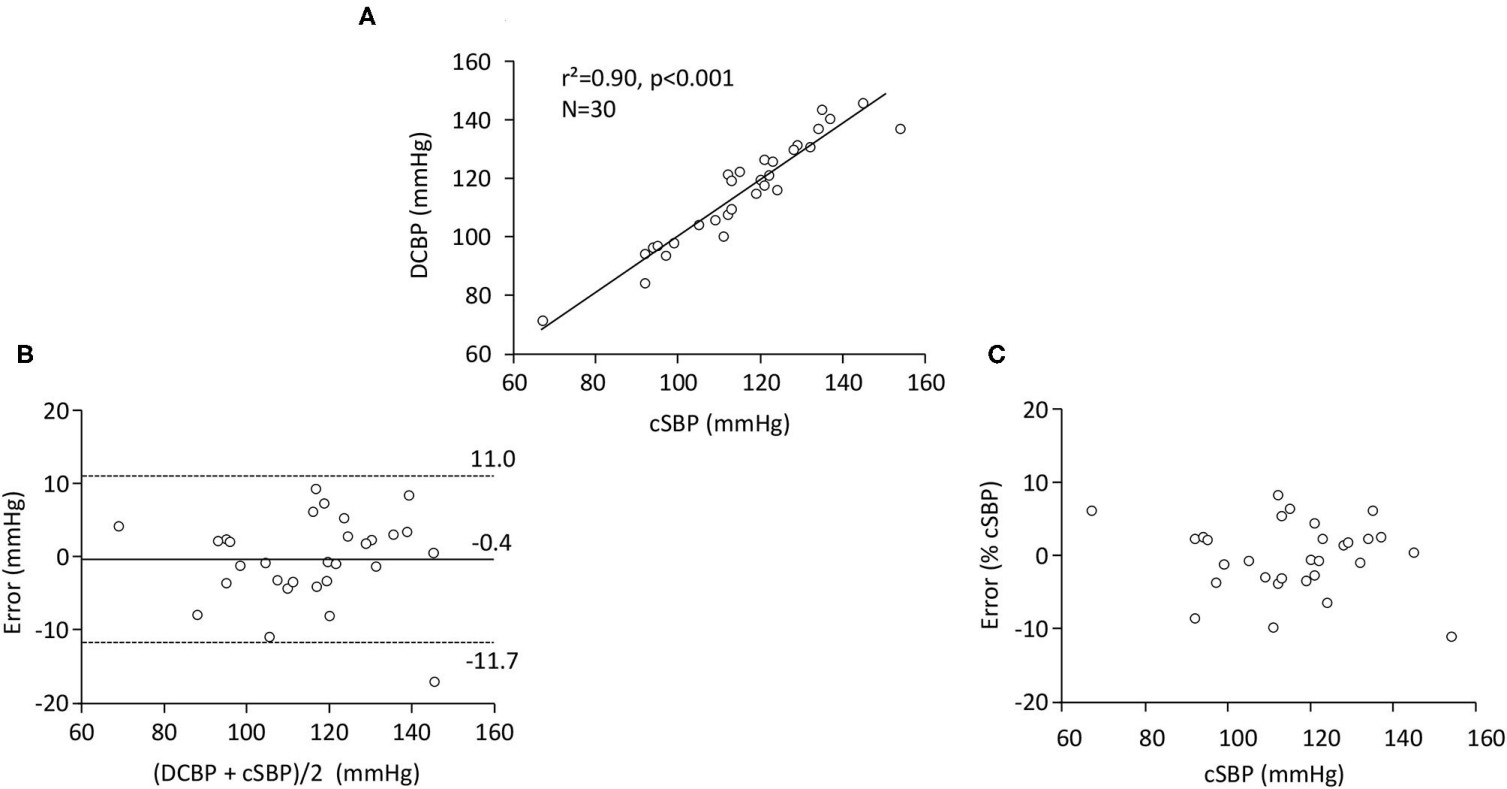
Carotid tonometry study (*n* = 30). **(A)** Linear relationship between the central systolic blood pressure (cSBP) estimated by carotid tonometry and DCBP calculated from radial MBP and DBP (fluid-filled catheter in the radial artery). **(B)** Bland and Altman plots of the error (DCBP-cSBP) as a function of average (DCBP+cSBP)/2). Dotted lines indicate 95% limits of agreement. **(C)** Influence of cSBP on the error (expressed as a percentage of cSBP).

There was a strong linear relationship between DCBP and cSBP [r^2^ = 0.90, p < 0.001 (Figure 3A)]. As compared to cSBP (115.7 ± 18.2 mmHg), DCBP gave an accurate estimate of cSBP (mean error = −0.4 mmHg) (Figure 3B). The precision was 5.8 mmHg and there was no bias across the range (r^2^ = 0.001, p = 0.8). The % error was −0.2 ± 4.8% of cSBP (range: from −11.1 to 8.2%) (Figure 3C). The number of comparisons falling within the 5, 10, and 15 mmHg error bands was 22 (73.3%), 28 (93.3%), and 29 (96.7%), respectively. The error was similar in men and women (p = 0.09), and was not influenced by heart rate (r^2^ = 0.11, p = 0.07) or by age (r^2^ = 0.06, p = 0.19).

### Sensitivity Analysis

Results of the sensitivity analysis are given in Table 4. Assuming a perfectly accurate non-invasive measurement of peripheral MBP as compared with intraarterial MBP (the 0% error column in Table 4), a positive/negative % error in the estimation of peripheral DBP will translate into a negative/positive % error of essentially similar magnitude for the cSBP estimation by the DCBP formula. Assuming a perfectly accurate non-invasive measurement of peripheral DBP (the 0% error line in Table 4), positive/negative % error in the estimation of peripheral MBP will translate into a positive/negative % error of roughly doubled value. The theoretical influences of combined calibration errors on the DCBP-derived estimation of cSBP are summarized in Table 4, with green areas indicating reasonable accuracy from a clinical standpoint (-10% to +10% error of cSBP using DCBP).

**Table 4:** Sensitivity analysis indicating the % error in the estimation of central systolic blood pressure (cSBP) by using the DCBP formula.

	**% error in the estimation of MBP**				
	**−10**	**−5**	**0**	**+5**	**+10**
% error in the estimation of DBP					
+10	−26	−18	−9	0	10
+5	−23	−14	−5	5	15
0	−19	−10	0	10	21
−5	−15	5	5	16	27
−10	−10	0	9	23	34

*The range of calibration error in the estimation of peripheral diastolic (DBP) and peripheral mean (MBP) blood pressure was set from −10% to +10%. DCBP = MBP^2^/DBP. Green areas indicate % error in cSBP estimation ≤ 10%. Red areas indicate % error in cSBP estimation > 10%. The % error indicated has been rounded up to the nearest whole number*.

## Discussion

Our work showed that cSBP may be estimated from peripheral MBP and DBP values only. The cSBP estimation is based on our new formula: DCBP = MBP^2^/DBP. As compared with methods currently used, there is no need for any supplementary device or waveform recording.

To evaluate any new method, it is important to identify the sources of errors which might impact the accuracy of the results (21, 22). For cSBP estimation, the potential sources of errors generally include the waveform acquisition technique (tonometry, oscillometry, and echo-tracking). This is irrelevant in our case as we did not rely on pulse acquisition. The method used to calibrate pulse waves is another potential source of error. Here, we based our new method on the widely admitted recommendation that MBP and DBP may be used as input factors for central BPs (rather than SBP and DBP) (21, 22, 25). The remaining sources of errors are: (1) the arterial site of pulse recording (radial, brachial, carotid, and aortic); (2) the method for BP measurement (invasive, non-invasive); and (3) the mathematical analysis used. For these reasons, we designed our study in three parts.

In the first part of our study, we focused on invasive high-fidelity aortic pressures, and the potential error due to mild differences between aortic and brachial MBP and DBP was excluded as only aortic BPs were considered. Thus, the main source of error tested was the mathematical analysis, i.e., the derivation of DCBP after having rearranged the geometric mean equation. We performed a *post-hoc* analysis of our previously published database (28). Over a wide cSBP range, the DCBP formula was associated with a−0.9 mmHg mean error and 7.4 mmHg SD of error, both of which fall within the guidelines of the Association for the Advancement of Medical Instrumentation (AAMI), namely <5 and <8 mmHg, respectively (36). However, one should remember that the AAMI guidelines have not been designed for cSBP validation. Our results are very similar to the pooled estimate of the mean error reported by Cheng et al. (21) and Papaioannou et al. (22) in two systematic reviews and metaanalyses of invasive central validation studies of commercial devices. Cheng et al. reported a mean error of −1.1 mmHg (95% CI: −9.1, 6.9 mmHg) (21) and Papaioannou et al. a mean error of −1.08 mmHg (95% CI: −2.81, 0.65 mmHg) (22). One advantage of DCBP is that the error in cSBP estimation was not influenced by the prevailing BP, unlike the way the input BP error impacts the transfer function output (27). The error was not associated with gender and was a slightly influenced by age and heart rate. Finally, the difference between DCBP and cSBP was felt within the 5, 10, and 15 mmHg error bands (34) in 62, 86, and 94% of the patients, respectively. A measurement error of 5 mmHg does not have the same diagnostic implication in hypotensive, normotensive, or hypertensive patients. Thus, it is also useful to express the error as a percentage of the measured value. The error between DCBP and the invasive central pressure was only - 0.4 ± 5.0% of cSBP. The interim conclusion of the first part of our study is thus that cSBP may be accurately and precisely estimated from central MBP and DBP values. Most of the commercial devices consider for granted that MBP and DBP only slightly differ from central aorta to peripheral large arteries and take advantage of this property to calibrate peripheral waveforms (1, 2, 21–27). Thus, a logical implication of our results is that cSBP may be accurately and precisely derived from high-quality invasive peripheral MBP and DBP values using the same DCBP formula (proof of concept).

In the second part of our study, we retrospectively analyzed patients from the literature in which high-fidelity pressures had been simultaneously obtained in the aorta and brachial artery (10, 11). The potential calibration error was related to the potential mild differences between brachial and central MBP and DBP. The accuracy of DCBP obtained from invasive high-fidelity brachial measurements (10, 11) was confirmed, with a weighed mean error of 1.1 mmHg (0.8%). As previously discussed (19), differences in MBP between aorta and brachial artery were of 0.4% in Kelly's et al. study (10) and of 0.1% in Sung's et al. study (11). The differences in DBP between aorta and brachial artery were of 3.1 and 1.4% in Kelly's et al. (10) and Sung's et al. (11) study, respectively. Four other high-fidelity pressure studies have independently documented differences in DBP between aorta and brachial artery of <2% (14–17). Overall, these results are in keeping with basic hemodynamic principles, especially the fact that the caliber of aorta and large arteries offers little resistance to blood flow (1, 20). One may conclude that these mild differences in MBP and DBP between aorta and brachial artery were responsible for a very mild extra-error in the cSBP estimation by DCBP. Since individual data were not available, the precision of the DCBP estimate could not be quantified.

In the third part of our work, we used carotid SBP as a non-invasive surrogate for cSBP, and invasive radial artery BPs obtained with “standard catheters” (i.e., fluid-filled instead of high-fidelity pressure catheters) as input factors. In this setting, measurement and calibration errors were more likely to impact accuracy and precision of DCBP. Due to its anatomical proximity and relatively similar characteristics, the carotid pressure is reported to be very similar to the aortic root pressure and often used as a non-invasive surrogate for central pressures (23, 33). As documented in the landmark book McDonald's Blood Flow in Arteries (20), carotid and aortic waveforms are similar when compared both in time domain and in the frequency domain. Carotid tonometry is also dependent on calibration; hence we used the same calibration from invasive radial DBP and the time-averaged radial MBP values to mitigate source of errors. Furthermore, Van Bortel et al. (34) have compared invasive cSBP measured at the aortic level and tonometry-obtained carotid waveforms. When calibrated to invasive aortic MBP and DBP, the non-invasive carotid cSBP was highly accurate compared with the invasive cSBP. In our work, DCBP calculated from invasive radial MBP and DBP provided accurate and precise estimation of cSBP. The DCBP formula was also stable across the BP range.

The high accuracy and reasonably good precision of DCBP may be explained by the fact that our study design minimized additional sources of error. First, when validating DCBP at the aortic level, we used data from high-fidelity pressure catheters as they must be preferred for validation studies (21–27). Although meticulously handled fluid-filled catheters may be acceptable and are currently used in clinical studies for practical and financial reasons, they are often associated with an overestimation of cSBP (24, 37–40). Secondly, when validating DCBP at the peripheral level, we used invasive peripheral BP recordings, as it has been reported, as this improves cSBP estimation (21–27). When calibration is performed using non-invasively measured peripheral BPs, two systematic reviews and meta-analyses of invasive central validation studies of commercial devices have consistently reported pooled estimates of the mean error of −8.2 mmHg (95% CI: −28.4, −12.0 mmHg) (21) and −5.81 mmHg (95% CI: −7.79, −3.84 mmHg) (22). This may be partly explained by the fact that whatever the non-invasive method used (oscillometric or auscultatory), DBP is overestimated by 6 mmHg and SBP is underestimated by 6 mmHg on average, as compared with intraarterial values (41). Mitchell also pointed out that the inaccuracy in peripheral pressure measurements with standard clinical tool (manual or automatic BP monitor) is in a range similar to the difference between central and peripheral SBP (7). Thirdly, the MBP value used in the DCBP formula was the most precise one, namely the time-averaged MBP.

The study has several strengths: (1) DCBP formula allows a simple and easy estimate of cSBP without requiring any supplemental device to record waveforms; and hence prevents the unavoidable technical error specifically and potentially associated with each type of device. (2) The proof of concept was established by studying 139 patients, which is a high number of patients for an invasive study, also bearing in mind that a *n* value >85 has been recommended for this type of validation study (25); and (3) the high accuracy and reasonably good precision of DCBP was observed over a wide cSBP range, with no bias across the range; and (4) DCBP was derived from a very simple and universally known mathematical formalism (the geometric mean), and this made it possible to quantify the proportion of input errors in MBP and DBP, which is transferred to the cSBP estimate when a non-invasive/less precise method of BP measurement will be used (Table 4). The field of applicability of DCBP may thus be predicted.

The limitations of our study must be also discussed. DCBP only estimated cSBP value. For this reason, artery tonometry or other waveform transfer function-based methods remain invaluable to estimate the entire pressure wave shape and to document other valuable arterial indices (e.g., augmentation index). Studied patients were mostly middle-aged or elderly men. Although the mean error was similar in men and women and only mildly influenced by age, further studies are needed in other populations to cover the overall spectrum of clinical conditions, especially in women and younger patients. The majority of the subjects entering the proof-of-concept study had their cSBP ≥ 140 mmHg at the time of the catheterization, although only one third had known hypertension. This may be explained by the mild sympathetic stimulation know to be often associated with left heart catheterization (37). It is likely that this does not alter the analysis and the results, but this point needs to be highlighted. The DCBP formula was further validated on a small ICU population and further studies are thus needed to confirm our results in a large, general population. It was not our aim to test the accuracy and precision of DCBP derived from non-invasive brachial BP recordings because we wished to minimize, as far as possible, measurement errors. Finally, and very importantly, the accuracy of the DCBP formula is dependent upon minimizing BPs measurement errors, but this is a limitation common to nearly all clinical devices aimed at non-invasively estimating cSBP. A reliable MBP value is especially needed, and it must be noted that the best empirical formula to estimate MBP from peripheral SBP and DBP is still under discussion and that the importance of this issue has been stressed (23, 24, 42–45).

The implications of our study should be discussed. The routine measurement of brachial BP is fundamental to assess the general health and cardiovascular risk of patients, including the diagnosis of hypertension and its subsequent management. The DCBP formula may help to easily estimate cSBP in clinics or help to assess the cardiovascular risk in patients from large clinical databases, both retrospectively and prospectively, provided measurement errors of MBP and DBP are minimized. The evidence supporting a tighter association of cardiovascular end points with central than peripheral BP remains controversial (3–9). This is a major issue because cardiovascular disease is the global number one cause of mortality. Factors limiting interpretation of available studies include the variety of technology used to estimate cSBP and the necessarily limited sample size (8). Applying DCBP could help to simplify and standardize the methodology and increase the sample size. From a hemodynamic point of view, a number of variables may also be derived from DCBP to gain deeper insight into the pathophysiology of cardiovascular diseases. This includes central pulse pressure (PP = DCBP – DBP), the double product (DCBP × heart rate), and left ventricular systolic pressure (in the absence of outflow tract obstruction) and its derived indices when coupled with echocardiography.

In conclusion, our study showed that cSBP could be estimated from peripheral MBP and DBP using DCBP (MBP^2^/DBP) in cases where BP measurement errors are minimized. This may have implications for assessing cardiovascular risk associated to cSBP on large BP databases, a point that deserves further studies.

## Data Availability Statement

The datasets presented in this article are not readily available because, data pertain strictly to Paris Hospitals. Requests to access the datasets should be directed to denis.chemla@aphp.fr.

## Ethics Statement

The studies involving human participants were reviewed and approved by the Ethics Committee of the French Intensive Care Society (agreement 12-376). The patients/participants provided their written informed consent to participate in this study.

## Author Contributions

DC: conception of the study and of the original DCBP formula, data acquisition, calculations, and writing. SM, OH, J-LT, XM, and FM: discussion of the results, analysis, and writing. MJ: data acquisition, calculations, and writing. All authors contributed to the article and approved the submitted version.

## Conflict of Interest

SM is the owner of Pulse Wave Consulting, a company providing consulting services to the medical devices industry such as Omron and MESI Ltd. This is outside the submitted work. OH had a paid lecture for Cheetah medical and is member of the scientific board of AMOMED. This is outside the submitted work. J-LT reports personal fees from Getinge. This is outside the submitted work. XM reports research grants, personal fees and non-financial support from Pulsion Medical Systems, and research grants and non-financial support from Baxter. This is outside the submitted work. FM is the founder and managing director of MiCo, a Swiss consulting and research company. This is outside the submitted work. The remaining authors declare that the research was conducted in the absence of any commercial or financial relationships that could be construed as a potential conflict of interest.

## Publisher's Note

All claims expressed in this article are solely those of the authors and do not necessarily represent those of their affiliated organizations, or those of the publisher, the editors and the reviewers. Any product that may be evaluated in this article, or claim that may be made by its manufacturer, is not guaranteed or endorsed by the publisher.
